# The Effect of Diet Supplementation with Pomegranate and Bitter Melon on Lipidomic Profile of Serum and Cancerous Tissues of Rats with Mammary Tumours

**DOI:** 10.3390/antiox9030243

**Published:** 2020-03-17

**Authors:** Agnieszka Białek, Małgorzata Jelińska, Małgorzata Białek, Tomasz Lepionka, Małgorzata Czerwonka, Marian Czauderna

**Affiliations:** 1 Department of Animal Improvement and Nutrigenomics, Institute of Genetics and Animal Breeding, Polish Academy of Sciences, Postępu 36A Jastrzębiec, 05-552 Magdalenka, Poland; 2 Department of Bromatology, Medical University of Warsaw, Banacha 1, 02-097 Warsaw, Poland; malgorzata.jelinska@wum.edu.pl (M.J.); malgorzata.czerwonka@wum.edu.pl (M.C.); 3 The Kielanowski Institute of Animal Physiology and Nutrition, Polish Academy of Sciences, Instytucka 3, 05-110 Jabłonna, Poland; m.bialek@ifzz.pl (M.B.);; 4 Laboratory of Hygiene, Food and Nutrition, Military Institute of Hygiene and Epidemiology, Kozielska 4, 01-163 Warsaw, Poland; tomasz.lepionka@wihe.pl

**Keywords:** CLA, CLnA, HETE, HODE, LOX, malondialdehyde, mammary tumour, oxysterol

## Abstract

The aim of this study was to present overall lipid profile of organisms with ongoing neoplastic process and applied diet supplementation with pomegranate seed oil (PSO) and bitter melon extract (BME). The following were quantified in serum and cancerous tissues of rats suffering from mammary tumours: fatty acids, conjugated fatty acids and sterols, their oxidised metabolites (malondialdehyde and oxysterols) and lipoxygenase (LOX) metabolites of polyunsaturated fatty acids. The obtained results indicate that abnormalities in lipid metabolism accompany neoplastic process. These differences concern all classes of lipids and most pathways of their transformation, with the special emphasis on lipid peroxidation and LOX-mediated metabolism. Cancer process appears to be so detrimental that it may conceal positive influence of dietary modifications. The lack of anticarcinogenic properties of PSO and BME in this model may be due to their antioxidant properties or elevated levels of conjugated linoleic acids (CLA), which change CLA isomer activity from anti- to pro-tumorigenic. As CLA are the product of conjugated linolenic acids (CLnA) endogenous metabolism, high CLA levels may be explained by applied diet enrichment.

## 1. Introduction

Since 1920, when Warburg observed that cancer cells show avid glucose uptake and tend to convert it to lactate through the glycolytic pathway regardless of whether oxygen is present (Warburg Effect) [1], many differences in metabolic pathways distinguishing cancer cells from normal cells have been described. Increased glutaminolysis [2], increased nucleotides synthesis [3] and abnormalities in lipid metabolism [4,5,6] are also recognised as characteristic features—hallmarks of cancer cells. Oncoproteins directly reprogram the metabolism of cancer cells and make them follow certain metabolic pathways [5]. It is generally considered that neoplastic process is characterised by metabolic reprogramming, which has been associated with cellular proliferation, energy storage and the generation of signalling molecules [7].

Uptake of lipoproteins and free fatty acids (FA) from the blood satisfy lipid requirements of most adult mammalian tissues, whereas de novo FA and cholesterol synthesis is restricted to a subset of tissues, including adipose tissue, liver and lactating breast [7,8]. Highly proliferating cancer cells either activate their endogenous de novo synthesis or increase the uptake of lipids and lipoproteins from bloodstream [4,9]. Many studies also reported an upregulated lipolysis and lipid oxidation in cancer cells, making FA oxidation a dominant bioenergetic pathway, e.g., in prostate cancer cells [5]. Tumour cells can also obtain indispensable FA by recommissioning them from their own structural and storage pools. Moreover, some of the most aggressive cancers can accumulate FA in intracellular lipid droplets [10]. Differences in lipid metabolism in ongoing neoplastic process are not limited to cancer cells themselves, but are also observed in surrounding tissues. Cachexia is a well-known effect of changes in lipid metabolism accompanying carcinogenesis, especially increased lipolysis in adipose tissue, well-recognised and observed in patients suffering from cancer [11]. Changes to lipid metabolism serve to create the most convenient microenvironment for cancer development.

Cholesterol, the main steroid compound in animal cells, is one of the basic components of their membranes, which maintains the stability of phospholipid bilayer. It is also present or even enhanced in membranes of multiple cancer cells, where it is the main component of lipid rafts [9]. Plasma levels of total cholesterol, triglycerides, high density lipoproteins (HDL) and low density lipoproteins (LDL) of breast cancer patients tend to be significantly higher than in control groups. It has been also established that cholesterol ester accumulation promotes breast cancer cell proliferation [12]. Hypercholesterolemia leads to an increased risk of breast, prostate and colon cancer. What is more, high consumption of dietary cholesterol increases the risk of breast cancer [13]. All of these corroborate cholesterol involvement in neoplastic process. Oxysterols are products of enzymatic and non-enzymatic oxidation of cholesterol, its precursors and phytosterols. They can be synthesised endogenously though different pathways, but they also are absorbed from diet, where their amount depends on temperature, oxygen, light exposure, lipid surrounding matrix and presence of antioxidants and water [14]. Oxysterols, which are present in physiological conditions in rather low amounts, are intermediates in cholesterol metabolism to bile acids and steroid hormones. They also act as lipid-signalling molecules with a wide range of properties, as they are ligands of some nuclear receptors (e.g., liver X receptors (LXR), estrogen receptors (ER)), G protein-coupled receptors (GPCR) and modulators of other receptors and proteins [13,15]. However, many researchers associate modified profile of oxysterols with numerous pathological conditions and diseases, e.g., neurological diseases or different types of cancer [15].

Oxidative stress, which is a result of imbalance in redox reactions, is characterised by enhanced production of reactive oxygen and nitrogen species (ROS and RNS, respectively). They can influence cell macromolecule structure and function. Lipid peroxidation, to which polyunsaturated FA (PUFA) are susceptible similarly as cholesterol, is one of the consequences of ROS action on cell membrane structure and function. Lipid hydroperoxides, which are primary products of PUFA peroxidation, are unstable and decompose to secondary products, such as malondialdehyde (MDA) or 4-hydroxynonenal (4-HNE), which in turn have prolonged half-life and can diffuse from their site of formation [16,17]. They serve as ‘oxidative stress second messengers’ or simply as ‘biomarkers of PUFA peroxidation’. ROS, primary and secondary products of PUFA peroxidation can participate in signal transduction, control of cell proliferation, induction of differentiation, maturation and apoptosis [17].

FA are not only the carriers of energy indispensable for cancer cells, but can also be used for the biosynthesis of an array of lipid-signalling molecules [11]. Eicosanoids, phosphoinositides, sphingolipids and certain FA are signalling lipids, whose accumulation alters the cellular biochemical foundation and might be a causal factor of malignant tumour progression and metastasis [4]. The term ‘eicosanoid’ refers to a group of oxygenated 20-carbon FA, which differ from other lipids in that they are essential, their precursors come solely from the diet and have an important role in cellular signalling [3]. Arachidonic acid (C20:4, c,c,c,c-Δ5,8,11,14, AA), which is one of the precursors for various eicosanoids, can be metabolised by different pathways providing diverse signalling molecules. The cyclooxygenase (COX) pathway leads to prostanoids (prostaglandins, thromboxanes and prostacyclins), the lipoxygenase (LOX) pathway leads to leukotrienes and hydroxyeicosatetraenoic (HETE) acids, whereas cytochrome P450 pathway provides the epoxyeicosatrienoic acids and hydroxyperoxyeicosatetraenoic acid [3]. Other PUFA also undergo LOX conversion creating different metabolites: linoleic acid (C18:2, c,c-Δ9,12, LA) provides hydroxyoctadecadienoic acids (HODE) and eicosapentaenoic acid (C20:5, c,c,c,c,c-Δ5,8,11,14,17, EPA) derivatives are hydroxyeicosapentaenoic acids (HEPE) [18].

Conjugated fatty acids (CFA) are geometric and positional isomers of PUFA with conjugated double bonds system in chain, which are characterised by health-promoting properties [19,20,21]. Due to similar structures to LA or α-linolenic acid (C18:3, c,c,c-Δ9,12,15, ALA), conjugated linoleic acids (CLA) and conjugated linolenic acids (CLnA) undergo analogous transformations to their unconjugated equivalents, interfere in their metabolism [22,23,24] and also influence COX and LOX metabolites formation. We previously indicated that CLA can influence HETE, HEPE and HODE formation in both physiological state and ongoing cancerous process [25,26,27]. We also demonstrated that dietary supplements such as pomegranate seed oil (PSO) - source of CLnA and bitter melon fruits aqueous extract (BME) interfere in FA metabolism and LOX metabolites formed in physiological state [18]. Due to lipid metabolism changes observed in neoplastic process, in the present study we wanted to check how diet supplementation with PSO and/or BME influences lipid metabolism in rats. That is why we decided to assess FA and sterol profiles, to quantify levels of their oxidised metabolites (MDA and oxysterols) and to check how these supplements will influence the levels of LOX metabolites in serum and mammary tumours. An overall lipidomic profile is expected to elucidate changes in lipid transformation appearing in cancerous tissue and its influence on general lipid status of the organism.

## 2. Materials and Methods

### 2.1. Bitter Melon Aqueous Extract (BME)

Fresh BME extract 1% (w/v) was prepared daily from bitter melon dried fruits, commercially available as the main ingredient of tea for brewing (Tra Kho Qua, Hung Phat Corp). Bitter melon tea was purchased from the local market in Warsaw, Poland. According to manufacturer’s description, measured quantity of hot water (80 °C) was added to the weighed portion of the dried material. The extract was filtered after 10 min, brought to ambient temperature and administered to animals ad libitum daily. Total characteristics of BME was given previously [28,29].

### 2.2. Pomegranate Seed Oil (PSO)

Cold pressed, unrefined PSO from seeds of pomegranate fruits (100%, ECOSPA), originating from Great Britain, was purchased from the local market in Warsaw, Poland. It was stored unopened at 8 °C in the original manufacturer’s dark glass package. Before administration to animals it was brought to ambient temperature and after that administered to the animals via gavage, in the amount of 0.15 mL per animal daily. Total characteristics of PSO was given previously [28,29].

### 2.3. Animals

A detailed description of the whole experiment was given previously [28]. The experiment and the guiding principles for the use and care of laboratory animals were approved by the II Local Ethical Committee on Animal Experiments in Warsaw (54/2015). Maiden Sprague-Dawley rats (*n* = 96, age 30 days) were purchased from Central Laboratory of Experimental Animals, Medical University of Warsaw. During the entire experiment they were kept in the animal room in plastic colony cages (three individuals per cage). Constant temperature (21 ± 1 °C) with 12 h light-dark cycle and relative humidity of 50–60% was maintained. Throughout the whole experiment animals were provided ad libitum access to a standard laboratory fodder Labofeed H (Feed and Concentrates Production Plant, A. Morawski, Żurawia 19, Kcynia, Poland) and drinking liquid. The detailed composition of Labofeed H fodder and applied dietary supplements was published previously [28]. After an adaptation period (7 days), rats were randomly divided into eight equinumerous groups. Their exact characteristics are given below:

- CON and CONplus—control groups without diet supplementation, fed a standard diet and water ad libitum,
- M and Mplus—animals fed a standard diet supplemented with 1% aqueous extract of bitter melon dried fruits ad libitum,
- G and Gplus—animals were fed the standard diet and water ad libitum and were given 0.15 mL/day pomegranate seed oil via gavage,
- GM and GMplus—animals were fed the standard diet and were supplemented with both 0.15 mL/day pomegranate seed oil administered via gavage and 1% aqueous extract of bitter melon dried fruits ad libitum.

The chemical carcinogen (7,12-dimethylbenz[a]anthracene (DMBA)) was applied to animals of groups marked with “plus”. DMBA dissolved in pomegranate seed oil (in case of Gplus and GMplus groups) or rapeseed oil (in case of CONplus and Mplus groups) was administered as solution via gavage in the dose 80 mg/kg body weight in the 50th day of life. During the entire experiment animals were daily monitored for any specific signs of welfare disorders (e.g., appetite loss, ruffling, sluggishness, apathy, hiding, curling up), palpated for tumour presence and weighed weekly. There were no spontaneous mammary tumours, nor other types of cancer in groups of animals not exposed to DMBA-treatment during the experiment. After 21 weeks of the experiment, rats from all groups were decapitated, exsanguinated and tumours were removed and weighed. Some randomly selected tumours were fixed in 10% formalin solution and subjected to histopathological examination, whereas the remaining tumours were stored in −80 °C for further analysis. A histopathological picture of tumours for CONplus, Gplus and Mplus groups corresponded to intraductal papillary adenoma, while tumours from the GM group corresponded to ductal adenoma [28].

### 2.4. Preparation of Research Material

Serum from DMBA-treated animals was obtained by centrifugation of blood for 10 min at 3000 rpm at 4 °C and stored at −70 °C until analysis. Only serum of animals suffering from mammary tumours was analysed. All mammary tumours obtained from DMBA-treated animals were stored deep-frozen in −80 °C for further analysis.

### 2.5. FA Analysis in Serum

FA gas chromatography (GC) analyses were performed only in serum samples obtained from individuals subjected to DMBA-treatment who developed mammary tumours during the experiment. Tumour-free animals from CONplus and Mplus groups were excluded. Analyses were performed with a gas chromatograph (GC-17A Shimadzu, Kyoto, Japan) equipped with a capillary column (BPX 70; 60 m × 0.25 mm i.d., film thickness 0.20 µm, SGE, Ringwood, Australia) and a flame-ionisation detection (FID). Detailed conditions were described previously [28]. FA methyl esters (FAME) standards (Supelco 37 Component FAME Mix, Sigma, St. Louis, MO, USA), CLA FAME reference standard (Nu-Chek-Prep, INC., Elysian, MN, USA), PA methyl ester reference standard (Methyl punicate, Matreya LLC, State College, PA, USA) were used to identify the FA present in samples. These standards were used to prepare standard curves for each FA and performed validation of the method. Results were expressed as µg of each FA per mL of serum and as a percentage share of each individual FA in total FA pool.

### 2.6. FA Analysis in Mammary Tumours

Detailed procedure of FA analysis was given previously [30]. Briefly, FA present in tumour samples were analysed using a gas chromatograph (GC) (GC-2010; SHIMADZU, Tokyo, Japan) coupled with a quadruple mass selective detector (MS) (Model 5973N; SHIMADZU, Tokyo, Japan); this chromatographic system was equipped with a BPX70 fused silica column (120 m × 0.25 mm i.d. × 0.25 μm film thickness; Phenomenex, Torrance, CA, USA), and an injection port, with helium as the carrier gas, according to Białek et al. [30], after derivatisation by the base- and acid-catalysed methylations procedures. FAME identification was validated based on electron impact ionisation spectra and compared to authentic FAME standards (Sigma, St. Louis, MO, USA) and the NIST 2007 reference mass spectra library (National Institute of Standard and Technology, Gaithersburg, MD, USA). All performed FAME analyses were based on total ion current chromatograms and/or selected-ion monitoring chromatograms. Nonadecanoic acid (C19:0) was used as an internal standard.

### 2.7. CFA Analysis in Mammary Tumours

Mammary tumour samples were subjected to alkaline hydrolysis according to the method of Czauderna et al. [31] prior to the chromatographic analysis. CFA isomers were analysed as free FA with Ag^+^-HPLC technique. Analysis was conducted on four analytical ion-exchange columns loaded with silver ions (Chrompack ChromSpher, 5 μm, Lipids, 250 × 4.6 mm; Varian, The Netherlands) and Waters HPLC 625LC system equipped with a photodiode array detector (DAD) (Milford, MA, USA) operated in a UV range from 195 to 400 nm. Sorbic acid (c2c4 C6:2, Sigma, St. Louis, MO, USA) was used as an internal standard and monitored at 259 nm [31]. CFA isomers were identified as conjugated dienes (CD) or conjugated trienes (CT) based on their retention times and UV spectra of analytical standards. Detection of CD analytical standards was conducted at 234 nm for C18:2, c,t-Δ9,11,-rumenic acid (RA) and C18:2, t,c-Δ10,12, (Larodan Fine Chemicals, Solna, Sweden). Detection of CT analytical standards was conducted at 270 nm for C18:3, t,t,c-Δ8,10,12,-α-calendic; C18:3, t,t,c-Δ9,11,13,-catalpic (CA); C18:3, c,t,t-Δ9,11,13,-α-eleostearic (ESA alpha); C18:3, t,t,t-Δ9,11,13,-β-eleostearic (ESA beta); and C18:3, c,t,c-Δ9,11,13,-punicic acid (PA) (Larodan Fine Chemicals, Solna, Sweden).

### 2.8. Malondialdehyde (MDA) Analysis in Mammary Tumours

For MDA content determination samples of mammary tumours were subjected to gentle alkaline saponification and derivatisation with 2,4-dinitrophenylhydrazine (DNPH) to form MDA-DNPH adducts [32]. A liquid chromatograph (UFLCXR system; SHIMADZU, Tokyo, Japan) equipped with C18-column (Synergi 2.5 μm, Hydro-RP, 100 Å, 100 × 2 mm, Phenomenex) and a DAD operated in the UV range of 195–420 nm was applied for MDA chromatographic analyses. As mobile phase linear binary gradient of acetonitrile in water was used. Solvent A consisted of water–acetonitrile (95:5, v/v) and solvent B of 100% acetonitrile. Identification of MDA-DNPH adducts peaks was based on retention time and absorption UV spectra of analytical standard—1,5-pentanedialdehyde (Sigma, St. Louis, MO, USA) solution (λ_max_ = 306 nm).

### 2.9. Total Cholesterol, Squalene and Oxysterol Analysis in Mammary Tumours

To mammary tumour samples 25 μL of 5α-cholestane (Sigma Aldrich, St. Louis, MO, USA) in chloroform (2 mg/mL) and 10 μL of butylhydroxytoluene in ethanol was added prior to homogenisation with 3 mL of KOH in ethanol solution (1 mol/L). Samples were incubated for 20 h in ambient temperature. Then, 4 mL of water and (2 × 2 mL) of hexane were added and samples were vigorously shaken. Combined hexane layers were dried in stream of N_2_. Afterwards 50 μL of pyridine and 25 μL of BSTFA with 1% TMCS (N,O-Bis(trimethylsilyl)trifluoroacetamide +1% of trimethylchlorosilane, Sigma Aldrich, St. Louis, MO, USA) were added and silylation procedure was performed at 80 °C for 45 min. Samples were brought to ambient temperature and 225 μL of hexane was added. Derivatised analytes were separated on GC-TOF/MS Pegasus^®^ BT (LECO Corporation, St. Joseph, MI, USA) chromatograph equipped with capillary column (30 m × 0.25 mm × 0.25 μm film thickness, Rxi^®^-17SilMS, Restek, Bellefonte, PA, USA) in splitless mode. The initial oven temperature was 200 °C for 4.6 min after that increased by 5 °C/min to 290 °C and held for 12.4 min. The injector was heated to 300 °C, the transfer line to 290 °C. Ion source temperature was 250 °C. Qualification was made on the basis of mass spectra and by comparison of retention times of analytes with these obtained for analytical standards (squalene, cholesterol, 7α-hydroxycholesterol, 7β-hydroxycholesterol, cholesterol 5α,6α-epoxide, 7-ketocholesterol; Sigma Aldrich, St. Louis, MO, USA) and quantification was based on external calibration curves prepared for analytical standards. 5α-cholestane (Sigma Aldrich, St. Louis, MO, USA) was used as an internal standard for recoveries.

### 2.10. LOX Metabolites Analysis in Serum and Mammary Tumours

HETE, HODE and HEPE were extracted from rat serum and mammary tumours with solid phase extraction method (SPE), described by Frohberg with some modifications [26,33]. Aliquots of 0.2–0.4 mL of serum samples were diluted with 0.5 mL of methanol and mixed vigorously. Water was then added to the samples to obtain about 10% methanol concentration. Subsequently, the specimens were loaded to the SPE C18 cartridges (BakerbondC18 500 mg/3 mL, J.T. Baker, Holland), conditioned previously with 10 mL of methanol and then 10 mL of water. The cartridges were washed with 2 mL of water followed by 2 mL of 10% methanol in water. Eicosanoids were eluted with 100% methanol (3 × 0.5 mL). The samples were evaporation to dryness under the stream of nitrogen and redissolved in 100 µL of ethanol.

HETE, HODE and HEPE were analysed by reversed-phase high performance liquid chromatography (HPLC) on the Shimadzu system consisted of LC-10AD pump, UV/VIS SPD-10AV detector and CTO-10AS column oven. Sample compounds were separated on the HPLC C18 column (Kinetex 2.6 μm, 100 × 4.6 mm, Phenomenex, Torrance, CA, USA) with the guard column filled with the same material. The analysis was carried out in a gradient system with the mobile phase consisted of two solvents: A (methanol/acetic acid, 100:0.01, v/v) and B (water with 0.01% acetic acid) at an initial ratio 70:30 (v/v). This ratio was maintained until 11 min and was changed to 27% B between 11.0 and 12.0 min and to 10% B between 18 and 19 min. Then the concentration of B phase remained unchanged to 25 min. when it was increased to the initial ratio of 30% that was held for 5 min. The flow rate was 0.8 mL/min and the column temperature was maintained at 35 °C. The detection wavelength was 235 nm [34]. The whole analysis lasted 30 min. The quantification of eicosanoids was based on external calibration curves prepared by triplicate injection of each of six dilutions of 15-, 12- and 5-HETE, 15-, 12- and 5-HEPE and HODE standards (Cayman Chemicals, USA).

### 2.11. Statistical Analysis

Data obtained are shown as mean values ± standard deviation (FA profile and content in serum, CFA percentage share in mammary tumours) and as median and min–max range (FA, CFA and oxysterols content in mammary tumours) due to the large differences among individual tumours in experimental groups. For variables with a skewed distribution, data were transformed into logarithms and retransformed after calculations. Results are presented as mean and confidence interval (marked *). All results were evaluated with Statistica12.5 (StatSoft, Cracow, Poland). Differences in examined variables among dietary groups were analysed with one-way ANOVA, with post-hoc HSD Tukey test. *p*-value ≤ 0.05 was considered significant.

In the summary section of the description of results, a linear discriminant analysis (LDA) was performed. In order to optimise LDA, the original data was transformed into natural logarithms and then standardised. Relevant discriminant functions were calculated in a stepwise progressive method, with the adopted tolerance value 1−*R^2^* = 0.01.

In order to identify the most changed metabolic pathways in the experiment, the metabolite set enrichment analysis (MSEA) was performed using a set of metabolites consisting of fatty acids and cholesterol and its derivatives. This analysis compared results from not-supplemented animals (CONplus) with PSO-supplemented groups (Gplus and GMplus). The MSEA used for analysis is an online tool available at http://www.msea.ca/MSEA/faces/Home.jsp. The Pathway Analysis module that was used combines results from powerful pathway enrichment analysis with pathway topology analysis to enhance the identification of the most relevant pathways involved in the studied conditions.

## 3. Results

### 3.1. FA in Serum

An overall FA profile of serum samples of animals suffering from mammary tumours was rather stable as total share of saturated FA (SFA), monounsaturated FA (MUFA) and PUFA did not differ among experimental groups (Table 1). PUFA were predominant in total FA pool. They constituted 45.9% of total FA on average, followed by SFA and MUFA, which constituted 33.0% and 11.5%, respectively. Among seven quantified SFA, significant differences among dietary groups referred to five of them. Diet supplementation with PSO increased the share of C14:0, C15:0 and C21:0 and decreased the share of C18:0. BME seems to affect C24:0 share (*p* = 0.0091), as in both BME supplemented groups a tendency to C24:0 increase was observed; however, HSD Tukey test did not reveal significant differences among groups. C16:1 was the only of four quantified MUFA whose level differed among investigated dietary groups (*p* = 0.0266) but post hoc test did not reveal any differences among investigated groups of animals. It suggests the opposing effect of PSO and BME, as in Gplus the share of C16:1 increased, whereas in GMplus it decreased. PSO influence was pronounced in terms to some n6PUFA, as it significantly increased LA and C20:2, c,c-Δ11,14, and decreased the AA share. This effect was stronger when PSO was applied to animals without BME extract. PSO also increased the levels of some of n3PUFA family: its precursor ALA and metabolites, such as EPA and DHA. No effect was observed for C20:3, c,c,c-Δ11,14,17, and DHA levels decreased. The overall n6PUFA share exceeded that of n3PUFA more than seven times but applied supplementation did not change this proportion. PA was not quantified in any of the examined samples, but C18:2, c,t-Δ9,11, which is a product of its conversion, was present in serum of all groups except Mplus. PSO supplemented to animals in pathological conditions did not increase RA share in total FA pool. As to absolute content of qualified FA in serum of rats, cancer process upset FA metabolism, which was reflected in a wide range of results among individuals within dietary groups. The influence of ongoing pathological process was so pronounced, that almost no differences in FA content were observed depending on the applied supplementation (Table A1).

### 3.2. LOX Metabolites in Serum

It was intended to determine seven compounds: 15-HETE, 12-HETE, 5-HETE, which are AA derivatives, HODE synthesised from LA and 15-HEPE, 12-HEPE and 5-HEPE belonging to EPA metabolites. However, EPA derivatives were not detectable in most serum samples. The ratios of compounds exerting opposite effects (12-HETE/15-HETE, 5-HETE/15-HETE) were also determined (Table 2). 12-HETE occurred in the highest concentrations of all the compounds tested in serum. Its content was the highest in the group supplemented simultaneously with PSO and BME, followed by the group fed exclusively with PSO. The lowest 12-HETE level was observed in BME supplemented rats. However, no statistically significant differences were observed. The other two AA metabolites were present in much lower concentrations than 12-HETE. 15-HETE ranged from 7.5 to 10.4 ng/mL. Similarly to 12-HETE, no significant differences in the levels of this eicosanoids were observed. The third of AA tested metabolites, 5-HETE, was found in the smallest concentrations of all detected eicosanoids. Its lowest level was observed in the Gplus group, followed by the GMplus and CONplus groups. The highest concentration of 5-HETE was determined in the BME supplemented group (Mplus group). Contrary to the other AA metabolites, the differences among 5-HETE levels were found to be significant (*p* < 0.0001). Significant differences were also found among the levels of HODE, LA derivatives. The highest content was observed in the M group. This result was significantly higher compared to all other groups. What is more, HODE concentration was also significantly increased in the Gplus group, supplemented with PSO, in comparison to the CONplus group.

12-HETE/15-HETE ratio was the highest in Gplus group and GMplus group. However, no significant differences were observed when compared to the control group and the M plus group. As to 5-HETE/15-HETE ratio, another pair of eicosanoids appearing to exert opposite effects, it was found to be the highest in the Mplus group. The results for Gplus and GM plus groups were significantly lower, compared to the Mplus group.

### 3.3. FA in Mammary Tumours

Mass spectrometry coupled with GC was applied as method of detection for FA analysis in mammary tumours. It allowed to quantify smaller amounts of FA in examined samples, which enlarged the spectrum of determined compounds. The total FA profile of cancerous tissue consisted of 12 SFA, 12 MUFA and nine PUFA (Table 3). As in serum, large variation in results within each dietary group and even among multiple tumours within a single individual were observed. Due to that the results were presented as median and the range (min–max) of values obtained for each experimental group. It manifested in several significant differences among a few FA. For three out of 12 SFA (C20:0, C22:0 and C24:0), differences were also observed. They concerned disparities between Gplus of the highest and GMplus of the lowest median of content. Only C24:1, c-Δ15 of MUFA exhibited significant differences between Mplus and Gplus groups. In case of AA, which was the sole FA whose levels differed between dietary groups among PUFA, its amounts were lower in Mplus and Gplus than in the GMplus group. Interestingly, despite the lack of significant differences in n3PUFA and n6PUFA absolute content, n6/n3 ratio was elevated in the Mplus group. No PA was incorporated into mammary tumours, but the product of its conversion, RA, was found in small amounts in both PSO supplemented groups.

### 3.4. CFA in Mammary Tumours

**Figure 1:**
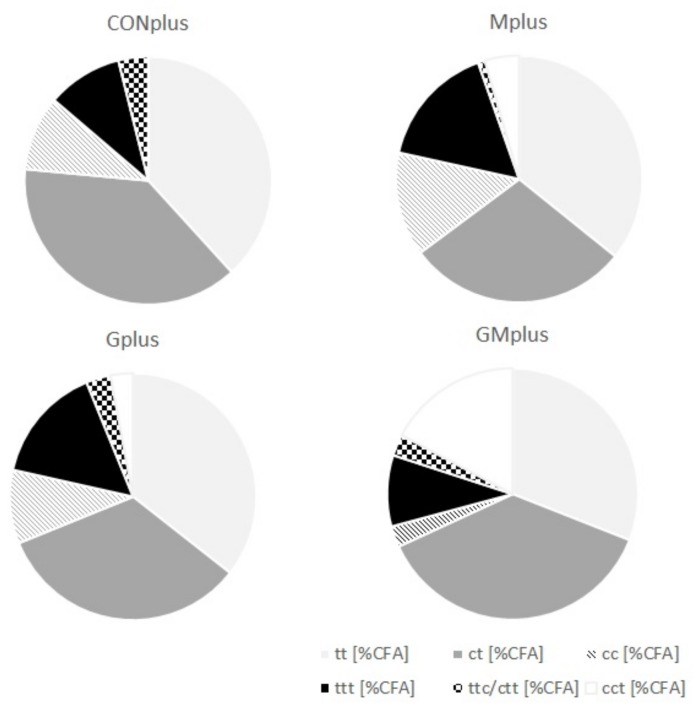
Detailed composition of different classes of conjugated fatty acid (CFA) isomers in mammary tumours of experimental groups.

Although GC-FID and GC-MS methods for determining FA profile in different biological samples are available, they seem to be insufficient for analysis of entire CFA profile. This is due to subtle structural differences among isomers, which make their separation and identification extremely difficult. The Ag+-HPLC-DAD method with analyses carried out on ion-exchange columns loaded with silver ions used in conjunction with a guard column containing the same stationary phase and photodiode array detection makes a good complement to the most commonly used GC methods. It enables good separation and differentiation of free FA containing two or three conjugated double bonds (CD—conjugated dienes and CT—conjugated trienes), which have different times of retention, UV spectra and the wavelengths of a maximum of absorption (λmax). CFA were quantified in cancerous tissues of all experimental groups and their content in both PSO supplemented groups was significantly elevated (Table 4). Both CD and CT were determined and their absolute content in tumours of Gplus and GMplus exceeded the content in CONplus and Mplus. The detailed composition of different classes of CFA isomers is presented in Figure 1. CD levels were higher than CT levels, constituting about 80% of CFA (Table 5). Among CD, share of *trans*,*trans* (tt) isomers was stable and amounted to 46% of CD, but their absolute content increased with PSO feeding meaningfully (*p* < 0.0001). The content of CD isomers with both *cis* bonds was elevated significantly in the Gplus group, but regarding their percentage share in total CD pool a decrease in GMplus was observed. The highest amounts of *cis*,*trans* (ct) CD isomers were quantified in pathological tissues of GMplus, although in Gplus they also exhibited a tendency to increase. However, percentage share of ct in total CD pool did not differ among experimental groups. c9t11CLA accounts for over 60% of all ct isomers (C18:2, c,t-Δ9,11), which corresponds to about 30% of total CD pool. The highest absolute amounts of c9t11CLA were determined in tumours of the GMplus group, which significantly exceeded their content in the Mplus group. CT were detected in cancerous tissues of all groups, but their amounts in both PSO fed groups exceeded those in remaining groups (*p* = 0.0003). Despite that, CT proportion in CFA did not differ among groups. The most abundant group of CT was *trans*,*trans*,*trans* (ttt) isomers, which constituted over 70% of all CT. Their elevated amounts were quantified in tumours of the groups obtaining PSO. Both percentage share and absolute amounts of two remaining groups of CT have not been modified by applied dietary supplements and *cis*,*cis*,*trans* (cct) isomers exceeded the *trans*,*trans*,*cis/ cis*,*trans*,*trans* (ttc/ctt) levels in all experimental groups.

### 3.5. LOX Metabolites in Mammary Tumours

**Figure A1:**
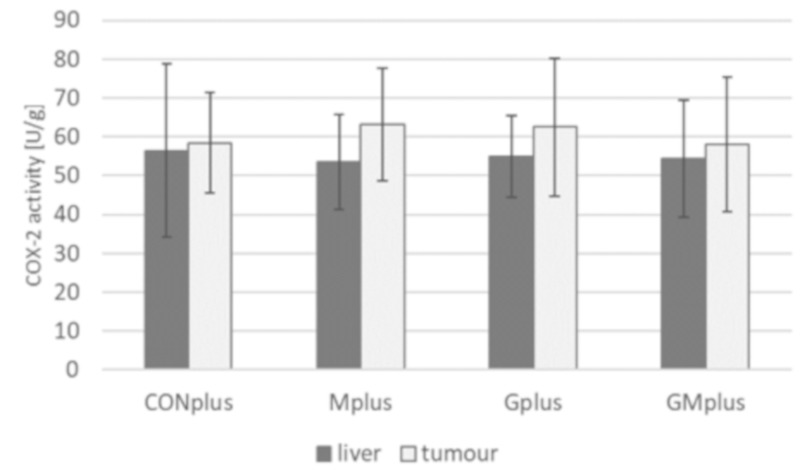
Cyclooxygenase (COX)-2 activity in livers and tumours of experimental groups.

COX-2 activity in livers and tumours of experimental groups did not differ (Figure A1). Due to this, only LOX metabolites were quantified. Extremely wide variations in eicosanoids levels in tumours in each dietary group were observed, so the results were presented as medians and concentration ranges (Table 6). EPA derivatives were not detected in tumour samples. Unexpectedly, among studied eicosanoids 15-HETE dominated in chemically induced tumours. Its highest level was observed in the Gplus group, followed by the GMplus group. However, these values were not significantly different in comparison to the control group and the Mplus group. Similarly to 15-HETE, HODE and 12-HETE levels were higher in the Gplus group compared to the control group and group supplemented with BME. For HODE, this was a statistically significant difference, compared to the Mplus group. No significant differences were also found among 12-HETE/15-HETE and 5-HETE/15-HETE ratios.

### 3.6. MDA Content in Mammary Tumours

**Figure 2:**
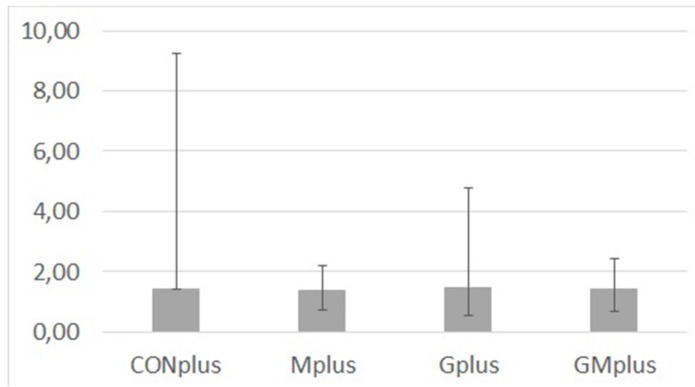
Malondialdehyde (MDA) content (µg/g of tissue) in mammary tumours of experimental groups.

Content of MDA in cancerous tissues of all experimental groups was diverse in individual tumours (Figure 2). Due to this, its amounts were presented as median and range. No significant differences were observed among dietary treatments, which indicates that ongoing cancerous process is a strong stressor that dysregulates the lipid oxidation processes.

### 3.7. Squalene, Cholesterol and Oxysterol Content in Mammary Tumours

In all samples of mammary tumours cholesterol precursor, squalene, and five oxidative metabolites (oxysterols) were quantified (Table 7). Amounts of all of them varied significantly between single tumours which resulted in lack of differences in examined parameters among dietary groups and indicated great dynamics of oxidation processes in cancerous tissues. The general claim was significantly increased ratio of cholesterol to squalene in tumours of the GMplus group and a similar tendency was observed for the Gplus group, which may indicate increased biosynthesis of cholesterol in these groups.

**Figure 3:**
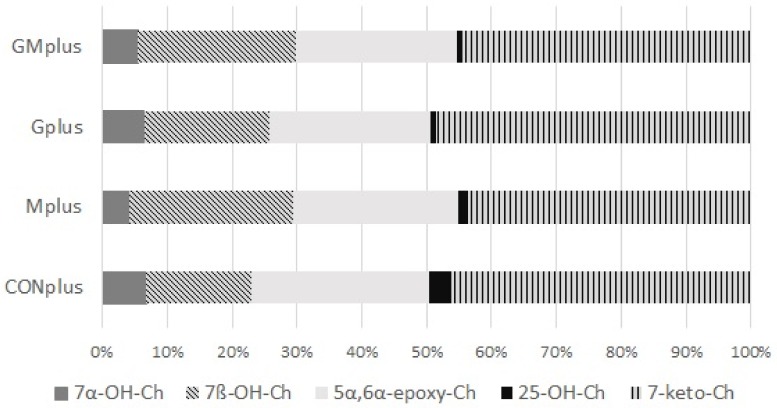
Oxysterols profile in mammary tumours of experimental groups.

Regarding oxysterols, their total amount in examined tumours did not differ among experimental groups. Similarly, the percentage share of oxysterols to cholesterol did not differ, although a decreasing tendency was observed in both PSO supplemented groups. Absolute amounts of all quantified oxysterols did not differ either among dietary groups. 7-keto-Ch predominated, followed by 5α,6α-epoxy-Ch and 7β-OH-Ch, whereas 7α-OH-Ch and 25-OH-Ch were present in smaller amounts. The percentage share of all quantified oxysterols in total oxysterols pool is presented in Figure 3. There were no significant differences in the percentage share of any of them in relation to applied dietary modifications.

### 3.8. Linear Discriminant Analysis (LDA)

Linear discriminant analysis (LDA) was used to obtain appropriate classification rules for examined tumour samples. A stepwise progressive method was used to calculate the discriminant functions as linear combinations of selected 25 descriptor values, related to the content of selected PUFA and indicators of oxidative process. In the applied method, initially all variables are outside the model. In the next steps the variables with the highest discriminant value, according to the Wilks’ λ statistic test, are included in the model. In our analysis, 19 variables were included in the final model. The canonical analysis allowed to distinguish three discriminatory functions (DF1, DF2 and DF3), which shown statistical significance in the Chi-square test (*p* < 0.0001). The coefficients of these functions and their average values are presented in Table A2.

The value of transferred variability indicates that the most significant function is DF1, as it explains over 74% of discriminatory power. The DF2 and DF3 explain 19% and 6.7% of discriminatory power, respectively. The analysis of canonical mean variables indicates that individual discriminatory functions significantly affect the distinction of specific groups. The analysis of these data combined with inspection of the charts of pairs of discriminatory functions allows to determine the contribution of individual functions to group differentiation.

**Figure 4:**
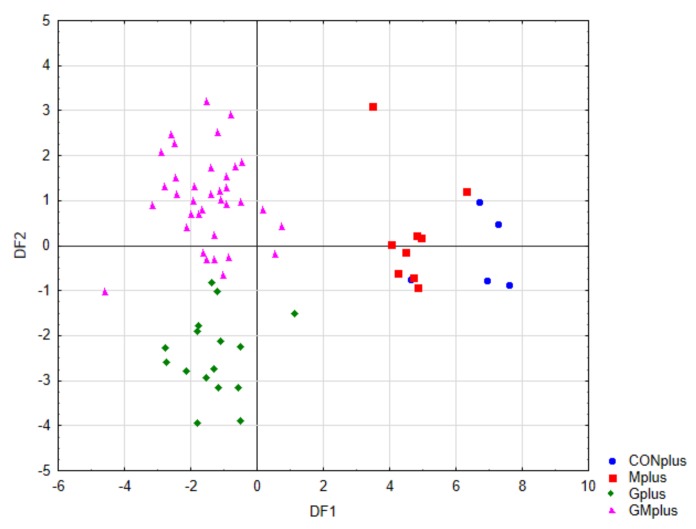
Scatterplot of canonical values for functions DF1 and DF2.

**Figure 5:**
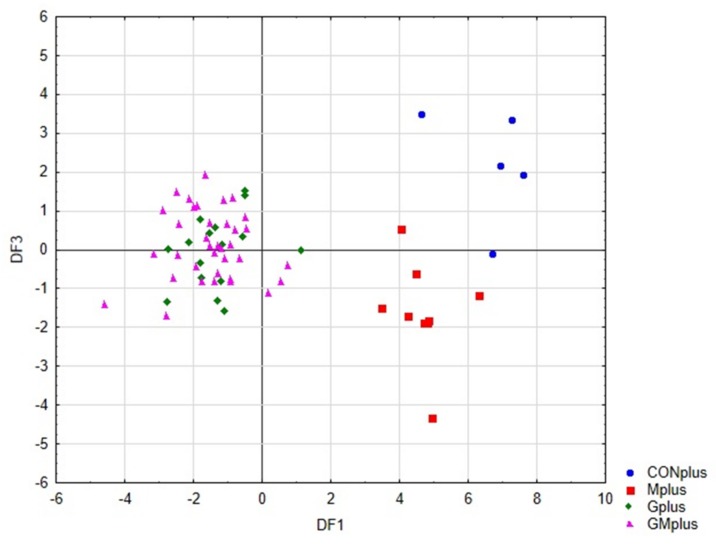
Scatterplot of canonical values for functions DF1 and DF3.

Figure 4 presents the scatter plot of canonical values for functions DF1 and DF2. A separation of all samples can be observed. The value of DF1, determined mainly by the content of conjugated dienes (tt, ct, including rumenic acid) and significantly affects the separation of PSO supplemented groups (Gplus and GMplus) from those not receiving this oil (CONplus and Mplus). In turn, DF2, determined mainly by the of individual LOX derivatives, clearly influences the separation of Gplus and GMplus groups. In Figure 5, presenting the scatterplot of the canonical values of the DF1 and DF3 functions, it can be observed that the separation of CONplus and Mplus groups depended on the value of DF3, which results mainly from the content of oxidised derivatives of cholesterol: 7ß-OH-Ch and 7α-OH-Ch.

Table 8 presents the classification efficiency matrix and the percentage predicted group membership for the original groups. These coefficients were highest for the Mplus and GMplus groups (100%), and their values for the Gplus and CONplus groups were 93.8% and 80%, respectively. Of the 66 cases, only two were incorrectly classified, which indicates a 96.9% classification efficiency of the proposed classification functions.

## 4. Discussion

Modification of lipid metabolism in cancer cells leads to structural changes in their membranes, disruption of their energy homeostasis, gene expression, cell signalling and protein distribution. These adjustments affect, in turn, numerous cell functions, such as growth, differentiation, proliferation, apoptosis, autophagy, necrosis, drug and chemotherapy resistance [35]. One of the most important metabolic hallmarks of cancer cells is enhanced lipogenesis. Cancer cells require a supply of lipids for proliferation and survival. The FA synthesis pathway is currently thought to be the major pathway exploited by cancer cells for the acquisition of lipids. In the presence of oxygen and abundant extracellular nutrients, most cancer cells synthesise FA de novo. Moreover, activation of de novo FA synthesis pathway is required for carcinogenesis [9]. Depending on the tumour type, tumour cells synthesise up to 95% of SFA and MUFA de novo in spite of sufficient dietary lipid supply [11]. However, under conditions of metabolic stress, scavenging for extracellular lipids is considered an important adaptive mechanism for cancer cells to maintain viability and/or growth [9]. Increased de novo synthesis as well as enhanced scavenging may resulted in lack of differences in SFA and MUFA content in mammary tumours derived from different dietary groups. We previously observed that ongoing neoplastic process influences FA profile in serum more than applied diet modification does [28]. The present data also confirm that the impact of cancer is so great that the effect of dietary modification on overall FA profile in serum is negligible and almost imperceptible, and is limited to individual FA of SFA, MUFA and MUFA. Moreover, overall content of FA in serum revealed dysregulation of lipid metabolism by ongoing neoplastic process. There are some significant differences in FA profile in breast adipose tissue in women with breast cancer and benign breast disease [36]. Other authors also observed significant differences in FA profile of serum, cancer tissue and surrounding tissues [37] as well as in red blood cell membranes [38]. FA content in cancerous cells reflects, to a great extent, FA content in their cell membranes, which in turn influences some important properties of cancer cells. Lewin et al. observed increased SFA and decreased MUFA in mammary adipose tissue of breast cancer in postmenopausal patients [39]. Increased content of SFA, which are packed more densely, changes membrane dynamics, which, in turn, makes the cancer cells more resistant to therapy as it limits the uptake of drugs [11]. Moreover, this excessive incorporation of FA into cancer cell membranes results in membrane phase separation, reduced cell–cell contact, and enhanced surface adhesion and tissue invasion [4], which in turn affects metastatic capacity. Large differences in FA content in cancer cells confirm dysregulation of lipid metabolism in the whole organism.

We have previously reported that increased content of RA in serum is connected with lower incidence of mammary tumours [26,40]. We also observed increased levels in serum and accumulation of CLA isomers in tissues as a result of diet enrichment with CLA; however, this tendency was diminished in pathological state [26,40,41,42,43]. Human study also indicated a reduced circulating CLA concentrations in breast cancer patients compared to controls [8]. It was also observed that PSO and BME elevated RA levels in serum in physiological state. This observation confirmed the ability of rats to metabolise CLnA into CLA isomers [28]. The present investigation confirmed differences in lipid metabolism in the pathological state, which are also reflected in differences in CLnA metabolism. Elevated levels of RA in serum after BME supplementation in the physiological state were not confirmed in individuals suffering from cancer. Moreover, its meagre share was below the limit of quantification (LOQ). Elevated levels of RA resulting from PSO supplementation were lower than previously observed in physiological state. CLnA metabolism in organisms with ongoing neoplastic process seems to be substantially changed making c9t11CLA and other CFA metabolites ineffective in diminishing cancer risk. Small levels of CLA isomers incorporated into cancer cell membranes after PSO supplementation in pathological conditions confirms this statement. Moreover, the high dose of CLA elicited mammary epithelial hyperplasia and enhanced tumorigenesis, whereas the low dose caused full inhibition of lipogenesis without undesirable proliferative and tumorigenic effects in mice [8]. It is supposed that diet supplementation with PSO resulted in too high levels of CLA, which in turn changed the way of its action from anti- to pro-tumorigenic. On the other hand, great similarity in the percentage share of particular classes of CFA isomers in mammary tumours of all experimental groups confirms almost identical remodelling of lipid metabolism in a pathological state, which is not susceptible to dietary interventions.

Some studies have reported high expression of COX-2 in breast cancer with no expression in normal breast tissue, whereas other studies have shown both upregulation and downregulation of COX-2 in some breast cancer as compared to unaltered breast tissue [3]. In present experiment no differences in COX-2 activity was observed among investigated dietary groups (Figure A1). LOX metabolites are also involved in physiological and pathological processes in organisms, especially when inflammation is an underlying cause, e.g., atherosclerosis, cardiovascular disease, diabetes, psoriasis or numerous types of cancer [44,45]. There is evidence that 12-HETE and 5-HETE promote cancer development and survival. Overexpression of 12-LOX and 5-LOX, enzymes converting AA to 12-HETE and 5-HETE, respectively, was observed in tumour cells, which in turn led to increased synthesis of these compounds. 12-HETE was observed to downregulate E-cadherin production and stimulate migration of endothelial cells [46]. It also increased angiogenesis and endothelial barrier permeability [47]. All these factors contribute to facilitated migration of tumour cells through the endothelial membrane and as a result to metastasis of cancer cells. 5-HETE was noted to act in a similar way, stimulating vascular endothelial growth factor (VEGF), one of the most potent tumour angiogenic factors, which resulted in angiogenesis promotion [48].

Some evidence indicates that diet, including dietary fat, may modify both circulating and tumour eicosanoid concentrations and as a result tumour incidence and growth [49,50]. Our previous studies also confirm that observation [25,26]. Initially they focused on the influence of CLA on HETE and HODE levels. Then we continued our investigations supplementing female rats with PSO and/or BME [18]. Both of them are dietary supplements popular worldwide and are potential dietary sources of CLnA. In the current study we went further by observing the influence PSO and BME on the content of eicosanoids under the conditions of pathological process such as cancer.

Similarly to most of our previous studies, 12-HETE was found in serum in the highest amounts of all the compounds tested. Its content was the highest in the group supplemented simultaneously with PSO and BME. Although the results did not differ significantly, they are in accordance with our previous observations in physiological state, without carcinogenic agent treatment [18]. 15-HETE and 5-HETE, the other two AA metabolites, were present in much lower concentrations than 12-HETE. The levels of 15-HETE were not statistically significant. As far as 5-HETE is concerned, it occurred in the smallest contents of all detected eicosanoids and unlike the other AA metabolites, the differences among 5-HETE levels among all the groups were found significant. Interestingly, this HETE isomer was not detected at all in our previous study where PSO and/or BME were administered, but rats were not treated with carcinogenic agents [18]. PSO contains considerable amounts of PA, belonging to CLnA, whereas bitter melon seeds are a source of α-eleostearic acid (60%), which is another isomer of CLnA, but only its trace elements may be detected in BME [28,29]. CLnA was found to be rapidly metabolised to CLA, which are considered to be active compounds. CLA isomers may influence LA metabolism by competing for the same enzymes. They seem to suppress Δ5-desaturase that converts C20:3, c,c,c-Δ8,11,14 to AA, a precursor of HETE (whereas C20:3, c,c,c-Δ,8,11,14 is synthesised from LA by Δ6-desaturase) [51]. RA and t10c12CLA (C18:2, t,c-Δ10,12) supplemented separately reduced Δ6-desaturation index in human serum. However, they did not affect the mRNA expression of desaturases and elongases, which may explain increased rather than diminished levels of 12-HETE in the groups supplemented with PSO and/or BME [52]. The highest levels of 15-HETE quantified in mammary tumours of all experimental groups were not observed previously for tumours of animals supplemented with CLA [26]. 15-LOX-1 and 15-LOX-2 are suggested to have tumour-suppressor roles in breast cancer, as their expression is reduced in malignant breast cells and tissues compared with healthy ones, whereas the expression of 5-LOXAP is increased in breast cancer and together with 12-LOX also associated with poor survival of the patient [3]. Elevated levels of 15-HETE in mammary tumours, which may result from 15-LOX-2 activity, were not connected with breast cancer incidence. This may indicate the smaller share of LOX metabolites in tumour development in this model.

As in our previous studies, 12-HETE/15-HETE and 5-HETE/15-HETE ratios were calculated, as pairs of eicosanoids, exerting potentially opposite effects. These factors were used as better indicators than particular HETE levels. Their decreased values correlated with CLA isomer supplementation and diminished tumour incidence. However, the current study did not confirm this observation and values of factors did not differ. Such a situation may be caused by intensive pathological process due to carcinogen administration to rats, which appears to influence and disturb many systems and functions, including eicosanoid synthesis.

Evidence from meta-analyses of Yang et al. [53] and Nindrea et al. [54] clearly confirms that higher intake ratio of n-3/n-6 PUFAs is associated with lower risk of breast cancer among females, which implies breast cancer prevention and treatment might be possible through increasing dietary intake ratio of n-3/n-6 PUFA. Additionally, Straka et al. [55] and Maillard et al. [56] observed increased accumulation of C20:5, c,c,c,c,c-Δ5,8,11,14,17 and C22:6, c,c,c,c,c,c-Δ4,7,10,13,16,19 in breast adipose tissue of women at high risk of breast cancer; the accumulation resulted from both diet supplementation and increased fish consumption. These results are in line with numerous studies demonstrating anticarcinogenic properties of long chain PUFA [36,57,58]. Novel preventive and therapeutic approaches include the attempts to use nanoparticle formulations for PUFA internalisation into cancer cells [59]. Lipids, particularly PUFA, are susceptible to oxidation by ROS, leading to lipid peroxidation that is harmful to cells. Lipid peroxidation triggers the propagation of lipid ROS that can significantly alter the properties of cell membranes or degrade into reactive compounds that cross-link DNA or proteins, exerting further toxic effect. Extensive lipid peroxidation can also result in oxidative stress-induced cell death [6]. Many authors claim that changes in lipid profile, especially increased levels of SFA, make cancer cells more resistant to oxidative stress-induced cell death [3,11]. Taking into account antioxidant properties of PSO and BME [28], levels of lipid peroxidation biomarkers were verified in mammary tumours. Great differences among individual tumours in each experimental group indicate distinctions and dysregulation of lipid peroxidation and are the reason for lack of significant differences in MDA levels. However, the highest maximal levels of MDA were detected in groups without BME supplementation, which indirectly seem to confirm antioxidant properties of BME and to a limited extent explains high incidence of mammary tumours. It was revealed that tumour cells are more resistant to lipid peroxidation than normal cells, which results from their PUFA deficiency caused by decreased activity of Δ5- and Δ6-desaturases [60]. Free radicals have both harmful and beneficial properties: in normal cells they can induce detrimental changes of macromolecules leading for example to neoplastic transformation, whereas in cancer cells they can activate apoptosis [16,60]. Gago-Dominguez, who proposed the hypothesis that lipid peroxidation represents a protective mechanism in breast cancer, also observed significantly lower MDA concentrations in plasma of patients with large tumours or in whom nodes and/or metastasis were present [17].

Oxysterols are products of oxidation of cholesterol mediated by cytochrome P450 enzymes. They are generated at a rate proportional to that of cholesterol synthesis. This group of compounds plays an important role in human cancer demonstrating the wide range of their possible involvement in neoplastic process: initiation and progression of cancer as well as beneficial influence of some oxysterols in certain types of cancer [13]. Oxidised metabolites of cholesterol interfere with cell proliferation by inhibiting it and can stimulate cell death by interfering with different pathways. It may result in cell death of various cancers, with no effect on senescent cells [13]. The lack of differences in oxysterol levels among dietary groups observed in present study is due to the large diversity of results obtained for individual tumours in each dietary group and also indicates dysregulation of lipid metabolism in cancer cells. There is also a great need for further studies employing genomic, proteomic and metabolomic profiling to further explain the role of oxysterols in carcinogenesis and their potential in cancer prevention or treatment.

In prostate cancer, cholesterol is a precursor of androgens and activates signalling pathways of cancer proliferation, promoting prostate cancer progression. Moreover, significantly higher total cholesterol and triglycerides levels were observed in breast cancer patients with metastasis than in those without lymphatic metastasis [12]. According to some authors, high levels of cholesterol might contribute to the metastases of prostate cancer into bones and simultaneous inhibition of sterol biosynthesis and intracellular cholesterol transportation may be an efficient way to treat breast cancer [13]. Despite the lack of significant differences in squalene and cholesterol levels in mammary tumours among dietary groups, elevated values of cholesterol to squalene ratio in both PSO supplemented groups seem to suggest enhanced synthesis of cholesterol and may contribute to elevated breast cancer incidence in these groups.

The linear discriminant analysis is a chemometric statistical method enabling identification of variables essential for group differentiation. It also allowed verification of differences of individual groups in multidimensional analysis, as well as development of a procedure to predict group membership for new cases. In this study, the applied LDA revealed significant differences between all experimental groups based on the selected variables concerning mostly the content of PUFA, CFA, LOX metabolites and indicators of oxidative status. A high value of the indicator of the average classification efficiency of the calculated functions indicates that the application of this method enables to define the origin of the tumour sample through analysis of selected parameters concerning lipid metabolism.

Metabolite set enrichment analysis (MSEA) was utilised to indicate which metabolic pathway may be the most affected by dietary supplementation with PSO in terms of mammary cancer development. The analysis identified the following metabolic pathways as most significant: primary bile acid biosynthesis, ALA metabolism, steroid hormone biosynthesis, LA metabolism, steroid biosynthesis and AA metabolism. Based on MSEA, the main observed differences in metabolic profiles of tumours were related to cholesterol and its derivatives (including oxysterols) levels. It confirms previous claims that elevated levels of cholesterol in both PSO supplemented groups contribute to elevated breast cancer incidence in these groups. Possibly, the increased proliferation rate of cancer cells requires fast supplementation of cholesterol and certain fatty acids.

## 5. Conclusions

Results obtained indicate great dysregulation of lipid metabolism accompanying ongoing neoplastic process. These differences concern all classes of lipids and most pathways of their transformation, with the special emphasis on lipid peroxidation and LOX-mediated metabolism. Cancer process appears to be so detrimental that its influence may conceal the influence of dietary modification. The lack of anticarcinogenic properties of PSO and BME in this model may be due to their antioxidant properties or elevated levels of conjugated linoleic acids (CLA), which change CLA isomers activity from anti- to pro-tumorigenic. As CLA are the product of conjugated linolenic acids (CLnA) endogenous metabolism, high CLA levels may be explained by applied diet enrichment.

## Figures and Tables

**Table 1:** Fatty acids (FA) percentage share in total FA pool in serum of experimental groups treated with chemical carcinogen (DMBA).

Fatty acids	CONplus	Mplus	Gplus	GMplus	*p*-Value
C14:0	0.24 ± 0.09 ^a,b^	0.30 ± 0.05	0.43 ± 0.10 ^a^	0.44 ± 0.06 ^b^	0.0052
C15:0	0.25 ± 0.06 ^a,b^	0.28 ± 0.03	0.65 ± 0.36 ^a^	0.50 ± 0.13 ^b^	0.0016
C16:0	16.0 ± 2.2	18.1 ± 2.9	19.6 ± 3.4	18.9 ± 2.4	n.s.
C17:0	0.64 ± 0.05	0.57 ± 0.13	0.68 ± 0.17	0.68 ± 0.13	n.s.
C18:0	17.2 ± 2.0 ^a,b^	13.7 ± 2.8	11.6 ± 3.1 ^a^	12.2 ± 1.6 ^b^	0.0125
C21:0	0.09 ± 0.01 ^a^	0.08 ± 0.01 ^b^	0.13 ± 0.04	0.15 ± 0.04 ^a,b^	0.0072
C24:0	0.41 ± 0.09	0.58 ± 0.03	0.41 ± 0.09	0.55 ± 0.09	0.0091
SFA	33.9 ± 1.4	33.8 ± 0.3	33.6 ± 1.2	33.6 ± 1.3	n.s.
C16:1	1.27 ± 0.70	1.14 ± 0.22	2.23 ± 1.15	1.60 ± 0.21	0.0266
C17:1, c-Δ10	0.24 ± 0.13	0.17 ± 0.04	0.37 ± 0.22	0.30 ± 0.10	n.s.
C18:1, c-Δ9	8.46 ± 1.91	7.23 ± 0.34	11.0 ± 3.7	9.42 ± 1.89	n.s.
C20:1, c-Δ11	0.07 ± 0.03	0.07 ± 0.03	0.09 ± 0.02	0.10 ± 0.03	n.s.
MUFA	10.1 ± 2.7	10.4 ± 2.9	13.7 ± 4.9	11.6 ± 2.2	n.s.
C18:2, c,c-Δ9,12 (LA)	11.1 ± 1.8 ^a,b^	11.9 ± 2.7	17.5 ± 2.8 ^a^	18.0 ± 2.4 ^b^	0.0022
6C18:3, c,c,c-Δ6,9,12 (GLA)	0.31 ± 0.02	0.36 ± 0.07	0.33 ± 0.06	0.33 ± 0.13	n.s.
C18:3, c,c,c-Δ9,12,15 (ALA)	0.28 ± 0.14 ^a^	0.25 ± 0.05	0.73 ± 0.30 ^a^	0.62 ± 0.20	0.0068
C18:2, c,t-Δ9,11 (RA)	0.05 ± 0.06	-	0.08 ± 0.02	0.12 ± 0.07	n.s.
C20:2, c,c-Δ11,14	0.07 ± 0.02 ^a^	0.10 ± 0.04	0.19 ± 0.10 ^a^	0.13 ± 0.06	0.0170
C20:3, c,c,c-Δ8,11,14	0.37 ± 0.04	0.29 ± 0.07	0.41 ± 0.06	0.44 ± 0.18	n.s.
C20:4, c,c,c-Δ5,8,11,14 (AA)	29.8 ± 4.2 ^a^	29.3 ± 2.4	19.5 ± 6.2 ^a^	21.1 ± 4.0	0.0127
C20:3, c,c,c-Δ11,14,17	0.27 ± 0.08	0.21 ± 0.00	0.39 ± 0.22	0.48 ± 0.38	n.s.
C20:5, c,c,c,c,c-Δ5,8,11,14,17 (EPA)	0.29 ± 0.13^a^	0.23 ± 0.07^b^	0.53 ± 0.15 ^a,b^	0.42 ± 0.08	0.0111
C22:2, c,c-Δ13,16	0.06 ± 0.02	0.10 ± 0.05	0.11 ± 0.04	0.07 ± 0.03	0.0383
C22:5, c,c,c,c,c-Δ7,10,13,16,19	0.35 ± 0.03 ^a^	0.44 ± 0.03	0.55 ± 0.19	0.63 ± 0.14 ^a^	0.0046
C22:6, c,c,c,c,c,c-Δ4,7,10,13,16,19 (DHA)	5.18 ± 0.97 ^a^	4.39 ± 0.32	3.21 ± 1.09 ^a^	3.65 ± 0.69	0.0123
PUFA	48.0 ± 3.2	46.1 ± 2.8	43.7 ± 5.4	45.8 ± 2.7	n.s.
n3PUFA	5.95 ± 0.66	5.10 ± 0.08	4.87 ± 1.03	5.35 ± 0.77	n.s.
n6PUFA	41.9 ± 2.7	40.8 ± 2.8	38.4 ± 4.8	40.5 ± 2.6	n.s.
n6/n3	7.08 ± 0.55	8.00 ± 0.60	8.09 ± 1.31	7.63 ± 1.11	n.s.

Data are shown as mean values ± standard deviation. Values in a row sharing a letter are statistically different. *p*-value ≤ 0.05 was considered significant. AA—arachidonic acid, ALA—α-linolenic acid, *c*—*cis,* DHA—docosahexaenoic acid, EPA—eicosapentaenoic acid, GLA—γ-linolenic acid, LA—linoleic acid, MUFA—monounsaturated fatty acids, n.s.—not significant, PUFA—polyunsaturated fatty acids, RA—rumenic acid, SFA—saturated fatty acids, *t*—*trans.* CONplus—control group without diet supplementation, fed a standard diet and water ad libitum, Mplus—animals fed a standard diet supplemented with 1% aqueous extract of bitter melon dried fruits ad libitum, Gplus—animals were fed the standard diet and water ad libitum and were given 0.15 mL/day pomegranate seed oil via gavage, GMplus—animals were fed the standard diet and were supplemented with both 0.15 mL/day pomegranate seed oil administered via gavage and 1% aqueous extract of bitter melon dried fruits ad libitum.

**Table 2:** Concentrations of linoleic and arachidonic acid lipoxygenase (LOX) metabolites (ng/mL) in serum of rats from experimental groups treated with chemical carcinogen (DMBA).

LOX Metabolites	CONplus	Mplus	Gplus	GMplus	*p*-Value
HODE	42.9 ± 2.6 ^a^	258 ± 37 ^a,b^	88.0 ± 10.5 ^a^	58.9 ± 5.1 ^b^	<0.0001
15-HETE	8.9 ± 1.2	10.4 ± 1.9	7.5 ± 1.0	8.3 ± 1.3	n.s.
12-HETE	316 ± 53	284 ± 41	391 ± 73	492 ± 89	n.s.
5-HETE	8.1 ± 0.6 ^a^	20.1 ± 2.4^b,c^	4.2 ± 0.4 ^a,b^	5.3 ± 0.9 ^c^	<0.0001
12-HETE/15-HETE ratio	38.5 ± 6.5	34.2 ± 5.8	55.2 ± 9.1	55.1 ± 10.3	n.s.
5-HETE/15-HETE ratio	1.9 ± 0.6	2.4 ± 0.4 ^a,b^	1.1 ± 0.4 ^a^	0.7 ± 0.1 ^b^	0.0081

Data were expressed as mean values ± standard error of mean. Values sharing a letter (a, b or c) in one row are significantly different (*p* ≤ 0.05); n.s.—not significant. CONplus—control group without diet supplementation, fed a standard diet and water ad libitum, Mplus—animals fed a standard diet supplemented with 1% aqueous extract of bitter melon dried fruits ad libitum, Gplus—animals were fed the standard diet and water ad libitum and were given 0.15 mL/day pomegranate seed oil via gavage, GMplus—animals were fed the standard diet and were supplemented with both 0.15 mL/day pomegranate seed oil administered via gavage and 1% aqueous extract of bitter melon dried fruits ad libitum.

**Table 3:** FA content (µg/g of tissue) in mammary tumours of experimental groups.

Fatty Acids	CONplus		Mplus		Gplus		GMplus		*p*-Value
	Median	Min–max	Median	Min–max	Median	Min–max	Median	Min–max	
ΣFA	7717	(3198–31,692)	10697	(3404–25,781)	4435	(3181–29,080)	7632	(3049–51,091)	n.s.
C8:0	14.5	(14.3–14.6)	8.34	(2.47–28.5)	11.6	(6.70–25.2)	10.2	(2.57–43.5)	n.s.
C10:0	29.7	(26.8–32.6)	6.83	(2.50–234)	13.8	(3.97–37.1)	12.6	(2.89–158)	n.s.
C12:0	19.5	(93.7–36.6)	16.5	(2.44–290)	18.4	(3.06–58.8)	19.9	(4.46–78.6)	n.s.
C14:0	85.8	(293–360)	139	(39.3–429)	67.1	(26.2–370)	122	(31.3–660)	n.s.
C15:0	30.3	(12.6–132)	33.7	(11.0–106)	20.8	(7.87–116)	28.8	(4.61–207)	n.s.
C16:0	1864	(834–7223)	2593	(942–7147)	1296	(725–7248)	2080	(736–12,240)	n.s.
C17:0	57.2	(26.0–264)	49.3	(23.8–191)	29.6	(11.8–177)	42.6	(13.6–363)	n.s.
C18:0	831	(559–1937)	855	(632–1555)	665	(521–1521)	786	(553–2850)	n.s.
C20:0	12.8	(9.15–27.4)	13.9	(6.81–30.1)	17.4 ^a^	(10.5–31.2)	13.3^a^	(3.55–45.1)	0.0427
C21:0	-		-		9.40	(1.39–16.8)	6.87	(1.87–12.5)	n.s.
C22:0	22.5	(18.5–24.5)	22.1	(12.6–28.5)	32.7 ^a^	(19.5–48.3)	8.03^a^	(1.35–57.6)	<0.0001
C24:0	19.2	(18.0–24.3)	21.1	(15.5–23.8)	25.3 ^a^	(16.8–42.5)	7.75^a^	(1.74–60.1)	<0.0001
SFA	2921	(1560–10,040)	3859	(1702–9096)	2058	(1528–9564)	3084	(1375–16,481)	n.s.
C16:1, c-Δ7	69.4	(26.9–278)	76.3	(39.4–169)	43.9	(20.1–194)	59.2	(19.2–394)	n.s.
C16:1, c-Δ9	191	(36.9–1353)	501	(49.7–2348)	137	(37.5–1665)	356	(47.6–3181)	n.s.
C17:1, c-Δ6	-		-		30.0	(27.4–43.8)	-		n.s.
C17:1, c-Δ9	-		-		68.2	(18.8–155)	133.4	(36.5–256)	n.s.
C18:1, t-Δ9	9.43	(5.53–24.5)	9.32	(3.34–24.0)	13.3	(6.37–25.4)	13.8	(0.77–50.3)	n.s.
C18:1, c-Δ6	8.82	(3.07–14.4)	7.10	(1.68–11.9)	11.0	(4.98–18.8)	8.58	(2.60–26.2)	n.s.
C18:1, c-Δ9	1838	(463–9832)	2720	(462–7024)	835	(409–8162)	1763	(462–14,577)	n.s.
C18:1, c-Δ11	313	(90.0–1583)	368	(90.4–910)	164	(83.9–1209)	252	(92.0–2262)	n.s.
C18:1, c-Δ12	9.66	(3.99–14.9)	8.76	(3.06–15.2)	-		8.63	(2.73–25.4)	n.s.
C18:1, c-Δ14	-		-		14.6	(6.59–17.5)	15.3	(1.28–29.1)	n.s.
C20:1, c-Δ11	21.2	(8.86–108.5)	38.4	(2.72–83.0)	28.0	(2.91–90.0)	21.0	(7.05–169.0)	n.s.
C24:1, c-Δ15	9.11	(5.82–23.0)	10.6^a^	(3.68–20.0)	32.3^a^	(6.62–49.6)	11.6	(0.95–41.3)	0.0447
MUFA	2457	(658–13,231)	3737	(656–10,542)	1282	(576–11,535)	2513	(642–20,012)	n.s.
C18:2, c,c-Δ9,12 (LA)	1331	(163–6677)	1734	(131–5943)	374	(150–6361)	768	(145–11,915)	n.s.
C18:3, c,c,c-Δ6,9,12 (GLA)	12.1	(2.70–49.1)	13.4	(3.11–19.3)	23.6	(3.77–44.0)	15.3	(7.48–49.5)	n.s.
C18:3, c,c,c-Δ9,12,15 (ALA)	180	(43.7–291)	77.2	(16.0–256)	90.4	(6.74–376)	64.9	(6.40–699)	n.s.
C18:2, c,t-Δ9,11 (RA)	-		-		53.2	(25.0–97.2)	33.6	(2.65–252)	n.s.
C20:2, c,c-Δ11,14	13.9	(4.51–51.3)	24.7	(6.47–41.5)	17.4	(6.14–46.6)	16.2	(2.39–78.3)	n.s.
C20:3, c,c,c-Δ8,11,14	28.5	(22.7–50.3)	33.3	(22.5–47.5)	40.3	(30.7–64.2)	37.2	(17.5–91.5)	n.s.
C20:4, c,c,c,c-Δ5,8,11,14 (AA)	828	(684–1050)	674^a^	(566–798)	704^b^	(479–993)	814^a,b^	(452–1946)	0.0035
C22:5, c,c,c,c,c-Δ7,10,13,16,19 (DPA)	18.0	(9.82–40.1)	22.5	(14.5–38.9)	28.7	(18.1–59.1)	27.1	(6.91–120.3)	n.s.
C22:6, c,c,c,c,c,c-Δ4,7,10,13,16,19 (DHA)	107	(75.7–186)	91.7	(65.1–144)	95.2	(51.6–161)	101	(61.7–323)	n.s.
PUFA	2329	(970 - 8395)	2584	(1040–7165)	1162	(945–7961)	1872	(818–14,530)	n.s.
n3PUFA	150	(85.6–517)	158	(107–439)	128	(91.7–567)	149	(78.9–1024)	n.s.
n6PUFA	2176	(885–7828)	2424	(933–6706)	1027	(854–7298)	1707	(727–13,205)	n.s.
n6/n3	12.6	(8.24–15.1)	13.9^a,b^	(8.73–17.7)	9.60^a^	(5.80–16.6)	11.4^b^	(6.48–15.3)	0.0160

Data are shown as median and the range of results (min–max). Values in a row sharing a letter are statistically different. *p*-value ≤ 0.05 was considered significant. AA—arachidonic acid, ALA—α-linolenic acid, *c*—*cis*, DHA—docosahexaenoic acid, DPA—docosapentaenoic acid, EPA—eicosapentaenoic acid, GLA—γ-linolenic acid, LA—linoleic acid, MUFA—monounsaturated fatty acids, n.s.—not significant, PUFA—polyunsaturated fatty acids, RA—rumenic acid, SFA—saturated fatty acids, *t*—*trans*. CONplus—control group without diet supplementation, fed a standard diet and water ad libitum, Mplus—animals fed a standard diet supplemented with 1% aqueous extract of bitter melon dried fruits ad libitum, Gplus—animals were fed the standard diet and water ad libitum and were given 0.15 mL/day pomegranate seed oil via gavage, GMplus—animals were fed the standard diet and were supplemented with both 0.15 mL/day pomegranate seed oil administered via gavage and 1% aqueous extract of bitter melon dried fruits ad libitum.

**Table 4:** CFA content (µg/g of tissue) in mammary tumours of experimental groups.

Conjugated Fatty Acids		CONplus		Mplus		Gplus		GMplus		
		Median	(Min–max)	Median	(Min–max)	Median	(Min–max)	Median	(Min–max)	*p*-Value
CFA		18.7 ^a,b^	(10.6–39.5)	23.8 ^c,d^	(17.0–60.5)	86.3 ^a,c^	(37.0–567)	104 ^b,d^	(27.2–954)	<0.0001
CD		15.5 ^a,b^	(8.48–37.2)	20.0 ^c,d^	(14.5–56.5)	65.3 ^a,c^	(29.7–544)	68.5 ^b,d^	(22.6–951)	0.0001
	tt	7.97 ^a,b^	(5.12–12.3)	9.50 ^c,d^	(6.28–19.4)	38.0 ^a,c^	(14.8–95.7)	30.0 ^b,d^	(12.4–154)	<0.0001
	ct	4.64	(2.17–24.8)	9.81	(2.21–28.1)	19.4	(2.15–433)	35.2	(2.80–774)	0.0192
	cc	2.93 ^a^	(0.47–3.41)	3.92 ^b^	(0.25–9.07)	10.0 ^a,b,c^	(3.33–32.1)	4.91^c^	(0.66–22.1)	0.0005
	c9t11CLA	3.74	(0.23–20.3)	5.94 ^a^	(0.62–20.9)	19.4	(2.15–391)	31.4 ^a^	(1.81–730)	0.0060
CT		2.15 ^a,b^	(1.21–3.22)	5.67 ^c^	(2.23–7.24)	16.0 ^a,c^	(2.78–32.4)	10.2 ^b^	(0.30–175)	0.0003
	ttt	1.56 ^a,b^	(0.30–2.29)	5.45 ^c^	(0.30–7.24)	15.8 ^a,c^	(3.37–32.4)	7.40 ^b^	(2.29–37.7)	<0.0001
	ttc/ctt	0.74	(0.51–1.21)	0.34	(0.34–0.34)	1.90	(0.43–3.29)	0.70	(0.12–26.4)	n.s.
	cct	-	-	0.87	(0.32–2.36)	1.68	(0.19–8.04)	9.26	(0.25–149)	n.s.

Data are shown as median and the range of results (min–max). Values in a row sharing a letter are statistically different. *p*-value ≤ 0.05 was considered significant. n.s.—not significant; CD—conjugated dienes, CFA—conjugated fatty acids, CLA—conjugated linoleic acid, c9,t11CLA—(C18:2, c,t-Δ9,11), CT—conjugated trienes, c—cis, t—trans. CONplus—control group without diet supplementation, fed a standard diet and water ad libitum, Mplus—animals fed a standard diet supplemented with 1% aqueous extract of bitter melon dried fruits ad libitum, Gplus—animals were fed the standard diet and water ad libitum and were given 0.15 mL/day pomegranate seed oil via gavage, GMplus—animals were fed the standard diet and were supplemented with both 0.15 mL/day pomegranate seed oil administered via gavage and 1% aqueous extract of bitter melon dried fruits ad libitum.

**Table 5:** CFA profile of mammary tumours of experimental groups.

Conjugated Fatty Acids		CONplus	Mplus	Gplus	GMplus	*p*-Value
CD [%CFA]		88.0 ± 7.2	82.2 ± 7.1	82.1 ± 11.1	79.2 ± 22.8	n.s.
	tt [%CD]	45.1 ± 13.7	45.5 ± 12.5	47.2 ± 18.4	48.4 ± 25.5	n.s.
	ct [%CD]	43.3 ± 17.6	37.4 ± 16.0	40.1 ± 22.5	50.5 ± 24.9	n.s.
	cc [%CD]	11.6 ± 6.7	17.1 ± 8.6 ^a^	13.6 ± 7.7 ^b^	4.06 (1.98–8.35) *^a,b^	0.0002
	c9t11CLA [%CD]	29.7 ± 20.5	24.8 ± 15.5	33.5 ± 21.0	41.2 ± 24.7	n.s.
	c9t11CLA [%ct]	61.2 ± 29.8	62.1 ± 20.1 ^a^	80.8 ± 22.8	83.7 ± 13.2 ^a^	0.0104
CT [%CFA]		12.0 ± 7.2	17.8 ± 7.1	17.9 ± 11.1	11.3 (3.3–38.5) *	n.s.
	ttt [%CT]	70.3 ± 3.7 ^a^	85.5 ± 17.4	86.5 ± 12.7	71.9 ± 30.2 ^a^	0.0394
	ttc/ctt [%CT]	43.8 ± 31.6	4.87 ± 0.01	13.7 (4.6–40.6) *	8.36 (2.52–27.7) *	n.s.
	cct [%CT]	-	42.2 ± 34.9	19.5 ± 13.1	57.0 ± 30.1	n.s.

Data are shown as mean values ± standard deviation. For variables with skew distribution data were transformed into logarithms and retransformed after calculations, and the results are presented as mean and confidence interval (marked *). Values in a row sharing a letter are statistically different. *p*-value ≤ 0.05 was considered significant. n.s.—not significant, CD—conjugated dienes, CFA—conjugated fatty acids, CLA—conjugated linoleic acid, c9,t11CLA—(C18:2, c,t-Δ9,11), CT—conjugated trienes, c—cis, t—trans. CONplus—control group without diet supplementation, fed a standard diet and water ad libitum, Mplus—animals fed a standard diet supplemented with 1% aqueous extract of bitter melon dried fruits ad libitum, Gplus—animals were fed the standard diet and water ad libitum and were given 0.15 mL/day pomegranate seed oil via gavage, GMplus—animals were fed the standard diet and were supplemented with both 0.15 mL/day pomegranate seed oil administered via gavage and 1% aqueous extract of bitter melon dried fruits ad libitum.

**Table 6:** Content of linoleic acid (LA) and arachidonic acid (AA) LOX metabolites (ng/g of tissue) in tumours of experimental groups.

LOX Metabolites	CONplus		Mplus		Gplus		GMplus		*p*-Value
	Median	Min–max	Median	Min–max	Median	Min–max	Median	Min–max	
HODE	354	(175–747)	312 ^a^	(217–1098)	688^a^	(295–2280)	627	(294–2398)	0.0140
15-HETE	575	(355–1684)	536	(258–1128)	879	(256 – 2182)	765	(284–2343)	n.s.
12-HETE	445	(270–539)	495	(236–2218)	637	(238–1075)	430	(144–6006)	n.s.
5-HETE	172	(62.0–569)	130	(80.9–576)	137	(59.4–362)	189	(34.6–710)	n.s.
12-HETE/15-HETE ratio	0.8	(0.3–0.9)	1.0	(0.4–8.6)	0.6	(0.3–1.5)	0.6	(0.3–3.9)	n.s.
5-HETE/15-HETE ratio	0.3	(0.2–0.6)	0.3	(0.1–1.2)	0.2	(0.1–0.4)	0.2	(0.3–0.9)	n.s.

Data are expressed as medians and ranges of results (min–max). Values sharing a letter in one row are significantly different (*p* ≤ 0.05), n.s.—not significant. CONplus—control group without diet supplementation, fed a standard diet and water ad libitum, Mplus—animals fed a standard diet supplemented with 1% aqueous extract of bitter melon dried fruits ad libitum, Gplus—animals were fed the standard diet and water ad libitum and were given 0.15 mL/day pomegranate seed oil via gavage, GMplus—animals were fed the standard diet and were supplemented with both 0.15 mL/day pomegranate seed oil administered via gavage and 1% aqueous extract of bitter melon dried fruits ad libitum.

**Table 7:** Contents of cholesterol, squalene and oxysterols (ng/mg of tissue) in mammary tumours of experimental groups.

Compounds	CONplus		Mplus		Gplus		GMplus		*p*-Value
	Median	(Min–max)	Median	(Min–max)	Median	(Min–max)	Median	(Min–max)	
squalene	112	(32.7–204)	71.7	(33.0–272)	62.8	(15.9–741)	47.3	(8.78–207)	n.s.
cholesterol	257	(10.6–561)	201	(3.81–1283)	409	(61.6–3867)	365	(12.0–1502)	n.s.
cholesterol/squalene	2.16^a^	(0.13–17.1)	2.99 ^b^	(0.10–7.60)	5.83	(0.33–43.6)	8.20 ^a,b^	(0.21–48.6)	0.0040
7α-OH-Ch	1.16	(0.28–5.93)	0.73	(0.20–1.35)	1.21	(0.53–8.14)	0.75	(0.16–6.06)	n.s.
7ß-OH-Ch	3.06	(1.00–4.17)	3.44	(2.00–9.32)	3.66	(2.37–54.6)	3.09	(2.09–20.0)	n.s.
5α,6α-epoxy-Ch	3.73	(3.22–13.3)	4.54	(1.66–6.63)	4.56	(2.86–77.1)	3.68	(2.54–13.0)	n.s.
25-OH-Ch	0.08	(0.06–3.27)	0.14	(0.11–1.22)	0.16	(0.06–4.56)	0.14	(0.06–0.47)	n.s.
7-keto-Ch	5.55	(3.60–38.9)	6.61	(2.51–20.4)	9.56	(3.21–140)	6.28	(2.11–65.5)	n.s.
Σ oxy-Ch	14.9	(8.29–62.2)	16.1	(8.39–32.1)	19.1	(9.17–276)	13.9	(7.92–88.2)	n.s.

Data are shown as median and the range of results (min–max). Values in a row sharing a letter are statistically different in post-hoc HSD Tukey test. *p*-value ≤ 0.05 was considered significant. n.s.—not significant;.7α-OH-Ch—7α-hydroxycholesterol, 7β-OH-Ch—7β-hydroxycholesterol, 5α,6α-epoxy-Ch—cholesterol 5α,6α-epoxide, 25-OH-Ch—25-hydroxycholesterol, 7-keto-Ch—7-ketocholesterol, oxy-Ch—oxysterols; CONplus – control group without diet supplementation, fed a standard diet and water ad libitum, Mplus—animals fed a standard diet supplemented with 1% aqueous extract of bitter melon dried fruits ad libitum, Gplus—animals were fed the standard diet and water ad libitum and were given 0.15 mL/day pomegranate seed oil via gavage, GMplus—animals were fed the standard diet and were supplemented with both 0.15 mL/day pomegranate seed oil administered via gavage and 1% aqueous extract of bitter melon dried fruits ad libitum.

**Table 8:** Classification results of linear discriminant analysis (LDA) presenting percentage predicted group membership for actual groups.

Actual Group	Number of Cases	Predicted Group Membership			
		CONplus	Mplus	Gplus	GMplus
**CONplus**	5	4 80.0%	1 20.0%	0 0%	0 0%
Mplus	9	0 0%	9 100%	0 0%	0 0%
Gplus	16	0 0%	0 0%	15 93.8%	1 6.2%
GMplus	36	0 0%	0 0%	0 0%	36 100%

CONplus—control group without diet supplementation, fed a standard diet and water ad libitum, Mplus—animals fed a standard diet supplemented with 1% aqueous extract of bitter melon dried fruits ad libitum, Gplus—animals were fed the standard diet and water ad libitum and were given 0.15 mL/day pomegranate seed oil via gavage, GMplus—animals were fed the standard diet and were supplemented with both 0.15 mL/day pomegranate seed oil administered via gavage and 1% aqueous extract of bitter melon dried fruits ad libitum.

## Abbreviations

4-HNE—4-hydroxynonenal; AA—arachidonic acid; ALA—α-linolenic acid; BME - bitter melon fruits aqueous extract; BSTFA—N,O-Bis(trimethylsilyl)trifluoroacetamide; CA—catalpic acid; CD—conjugated dienes; CFA—conjugated fatty acids; CLA—conjugated linoleic acids; CLnA—conjugated linolenic acids; COX—cyclooxygenase; CT—conjugated trienes; DHA—docosahexaenoic acid; DPA—docosapentaenoic acid; DMBA—7,12-dimethylbenz[a]anthracene; DNPH—2,4-dinitrophenylhydrazine; EPA—eicosapentaenoic acid; ESA alpha—α-eleostearic acid; ESA beta—β-eleostearic acid; FA—fatty acids; FID—flame-ionisation detection; GC—gas chromatography; GLA—γ-linolenic acid; GPCR—G protein-coupled receptors; HDL – high density lipoprotein; HEPE—hydroxyeicosapentaenoic acids; HETE—hydroxyeicosatetraenoic acids; HODE—hydroxyoctadecadienoic acids; HPLC—high performance liquid chromatography; LA—linoleic acid; LDA—linear discriminant analysis; LDL – low density lipoprotein; LOQ—limit of quantification; LOX—lipoxygenase; MDA—malondialdehyde; MS—mass spectrometry; MUFA—monounsaturated fatty acids; PA—punicic acid; PSO—pomegranate seed oil; PUFA—polyunsaturated fatty acids; RA—rumenic acid; RNS—reactive nitrogen species; ROS—reactive oxygen species; SFA—saturated fatty acids; SPE—solid phase extraction; TMCS—trimethylchlorosilane.

## Appendix A

**Table A1:** FA content (µg/mL) in serum of experimental group treated with chemical carcinogen (DMBA).

Fatty Acids	CONplus	Mplus	Gplus	GMplus	*p* Value
C14:0	2.79 ± 1.27	3.04 ± 1.08	5.64 ± 3.89	5.05 ± 2.48	n.s.
C15:0	4.53 ± 0.92	4.84 ± 1.66	8.35 ± 3.88	7.34 ± 2.41	n.s.
C16:0	481 ± 207	628 ± 205	606 ± 251	524 ± 137	n.s.
C17:0	15.3 ± 6.9	14.7 ± 6.0	16.7 ± 7.7	14.9 ± 4.9	n.s.
C18:0	441 ± 181	386 ± 50	292 ± 123	283 ± 104	n.s.
C21:0	0.99 ± 0.20	1.82 ± 2.90	0.78 ± 0.56	1.45 ± 0.85	n.s.
C24:0	8.86 ± 4.03	18.8 ± 11.7	7.06 ± 2.21	9.32 ± 2.87	n.s.
SFA	951 ± 390	1057 ± 215	937 ± 298	845 ± 234	n.s.
C16:1	23.3 ± 14.7	22.4 ± 0.6	45.7 ± 40.5	30.4 ± 13.5	n.s.
C17:1, c-Δ10	4.84 ± 1.75	2.56 ± 1.23	8.07 ± 5.82	4.79 ± 2.66	n.s.
C18:1, c-Δ9 (OL)	246 ± 124	288 ± 160	339 ± 221	249 ± 119	n.s.
C20:1, c-Δ11	1.02 ± 0.47	0.49 (0.09-2.78) *	1.44 ± 1.12	1.23 ± 0.80	n.s.
MUFA	274 ± 138	314 ± 163	387 ± 262	285 ± 133	n.s.
C18:2, c,c-Δ9,12 (LA)	351 ± 166	561 ± 299	566 ± 174	527 ± 131	n.s.
C18:3, c,c,c-Δ6,9,12 (GLA)	5.32 ± 2.82	6.98 ± 0.53	7.85 ± 7.42	4.73 ± 2.60	n.s.
C18:3, c,c,c-Δ9,12,15 (ALA)	5.31 ± 2.10	6.87 (2.44–9.32) *	14.9 ± 10.6	11.9 ± 6.96	n.s.
C18:2, c,t-Δ9,11 (RA)	-	-	0.46 ± 0.01	0.52 ± 0.01	n.s.
C20:2, c,c-Δ11,14	0.42 ± 0.17	0.97 (0.35–2.66) *	1.82 (0.57–5.81) *	1.55 ± 1.14	n.s.
C20:3, c,c,c-Δ8,11,14	6.57 ± 2.52	6.28 ± 2.77	8.73 ± 3.41	7.06 ± 3.00	n.s.
C20:4, c,c,c,c-Δ5,8,11,14 (AA)	914 ± 451	784 ± 172	564 ± 233	572 ± 187	n.s.
C20:3, c,c,c-Δ11,14,17	5.19 ± 2.28	4.69 ± 0.10	2.55 (0.67–9.67) *	8.68 (1.49–50.7) *	n.s.
C20:5, c,c,c,c,c-Δ5,8,11,14,17 (EPA)	5.90 ± 3.68	4.88 ± 1.24	11.3 ± 4.5	10.3 ± 8.1	n.s.
C22:2, c,c-Δ13,16	1.07 ± 0.44	2.01 ± 0.96	2.19 ± 0.82	1.02 ± 0.23	0.0100
C22:5, c,c,c,c,c-Δ7,10,13,16,19	12.2 ± 6.3	20.8 ± 5.2	18.4 ± 3.5	20.1 ± 5.3	n.s.
C22:6, c,c,c,c,c,c-Δ4,7,10,13,16,19 (DHA)	194 ± 101	178 ± 20	115 ± 49	122 ± 45	n.s.
PUFA	1499 ± 707	1576 ± 180	1315 ± 333	1314 ± 325	n.s.
n3PUFA	208 ± 104	194 ± 32	146 ± 57	180 ± 91	n.s.
n6PUFA	1290 ± 606	1379 ± 148	1165 ± 281	1131 ± 281	n.s.
n6/n3	6.23 ± 0.71	7.14 ± 0.43	8.49 ± 1.89	7.18 ± 2.17	n.s.

Data are shown as mean values ± standard deviation. For variables with a skewed distribution, data were transformed into logarithms and retransformed after calculations and results are presented as mean and confidence interval (marked *). *p*-value ≤ 0.05 was considered significant. AA—arachidonic acid, ALA—α-linolenic acid, *c*—*cis*, DHA—docosahexaenoic acid, EPA – eicosapentaenoic acid, GLA—γ-linolenic acid, LA—linoleic acid, MUFA—monounsaturated fatty acids, n.s.—not significant, PUFA—polyunsaturated fatty acids, RA—rumenic acid, SFA—saturated fatty acids, *t*—*trans*.

**Table A2:** Coefficients and average value of canonical variables included in the final model.

Coefficients of Canonical Variables			
Variable (Discriminatory Power)	DF1 (74.3%)	DF2 (19.0%)	DF3 (6.7%)
tt	−2.87486	−0.23823	0.84560
cc	0.41445	−0.88030	−0.61785
tumour mass	0.46920	−0.81715	−0.51740
ct	3.27553	0.21526	0.24013
ttt	−0.15933	−0.65019	−0.23386
7α-OH-Ch	−0.53719	−0.62480	0.20176
5-HETE/15-HETE	−0.38686	2.92710	−0.12543
HODE	−1.59109	0.16220	0.67288
12-HETE/15-HETE	1.21217	−2.15447	−0.25894
MDA	1.03007	−0.39936	−0.05724
squalene	0.85398	−0.66452	0.73374
7ß-OH-Ch	0.03831	0.24962	−1.68921
RA	−2.05800	0.10286	−0.65535
5α,6α-epoxy-Ch	−0.44120	0.35613	1.36723
15-HETE	1.44316	−0.55230	−0.08770
12-HETE	−0.58887	1.98339	−0.45563
5-HETE	−0.02018	−2.08316	0.29005
25-OH-Ch	0.40469	−0.10663	−0.07427
cholesterol/squalene	−0.34923	0.20157	−0.12313
**Average value of canonical variables**			
CONplus	6.63668	−0.19222	2.16305
Mplus	4.64837	0.27063	−1.58810
Gplus	−1.31511	-2.42687	−0.04219
GMplus	−1.49936	1.03765	0.11535

c—cis, RA—rumenic acid, t—trans, 7α-OH-Ch—7α-hydroxycholesterol, 7β-OH-Ch—7β-hydroxycholesterol, 5α,6α-epoxy-Ch—cholesterol 5α,6α-epoxide, 25-OH-Ch—25- hydroxycholesterol, 7-keto-Ch—7-ketocholesterol. CONplus—control group without diet supplementation, fed a standard diet and water ad libitum, Mplus—animals fed a standard diet supplemented with 1% aqueous extract of bitter melon dried fruits ad libitum, Gplus—animals were fed the standard diet and water ad libitum and were given 0.15 mL/day pomegranate seed oil via gavage, GMplus—animals were fed the standard diet and were supplemented with both 0.15 mL/day pomegranate seed oil administered via gavage and 1% aqueous extract of bitter melon dried fruits ad libitum.
